# *ABCA4* c.6480-35A>G, a novel branchpoint variant associated with Stargardt disease

**DOI:** 10.3389/fgene.2023.1234032

**Published:** 2023-09-07

**Authors:** María Rodríguez-Hidalgo, Suzanne E. de Bruijn, Zelia Corradi, Kim Rodenburg, Araceli Lara-López, Alicia Valverde-Megías, Almudena Ávila-Fernández, Lidia Fernandez-Caballero, Marta Del Pozo-Valero, Jordi Corominas, Christian Gilissen, Cristina Irigoyen, Frans P. M. Cremers, Carmen Ayuso, Javier Ruiz-Ederra, Susanne Roosing

**Affiliations:** ^1^ Department of Neuroscience, Biodonostia Health Research Institute, Donostia-San Sebastián, Spain; ^2^ Department of Genetic, Physical Anthropology and Animal Physiology, University of the Basque Country UPV/EHU, Leioa, Spain; ^3^ Department of Human Genetics, Radboud University Medical Center, Nijmegen, Netherlands; ^4^ Miramoon Pharma S.L., Donostia-San Sebastián, Spain; ^5^ Ophthalmology Service, San Carlos Clinical Hospital of Madrid, Madrid, Spain; ^6^ Department of Genetics, Health Research Institute-Fundación Jiménez Díaz University Hospital, Universidad Autónoma de Madrid (IIS-FJD, UAM), Madrid, Spain; ^7^ Center for Biomedical Network Research on Rare Diseases (CIBERER), Instituto de Salud Carlos III, Madrid, Spain; ^8^ Radboud Institute of Molecular Life Sciences, Radboud University Medical Center, Nijmegen, Netherlands; ^9^ Ophthalmology Service, Donostia Universy Hospital, Donostia-San Sebastián, Spain; ^10^ Department of Ophthalmology, University of the Basque Country (UPV/EHU), San Sebastián, Spain

**Keywords:** branchpoint variant, midigene splice assay, whole genome sequencing, *ABCA4*, Stargardt disease

## Abstract

**Introduction:** Inherited retinal dystrophies (IRDs) can be caused by variants in more than 280 genes. The ATP-binding cassette transporter type A4 (*ABCA4*) gene is one of these genes and has been linked to Stargardt disease type 1 (STGD1), fundus flavimaculatus, cone–rod dystrophy (CRD), and pan-retinal CRD. Approximately 25% of the reported *ABCA4* variants affect RNA splicing. In most cases, it is necessary to perform a functional assay to determine the effect of these variants.

**Methods:** Whole genome sequencing (WGS) was performed in one Spanish proband with Stargardt disease. The putative pathogenicity of c.6480-35A>G on splicing was investigated both *in silico* and *in vitro*. The *in silico* approach was based on the deep-learning tool SpliceAI. For the *in vitro* approach we used a midigene splice assay in HEK293T cells, based on a previously established wild-type midigene (BA29) containing *ABCA4* exons 46 to 48.

**Results:** Through the analysis of WGS data, we identified two candidate variants in *ABCA4* in one proband: a previously described deletion, c.699_768+342del (p.(Gln234Phefs*5)), and a novel branchpoint variant, c.6480-35A>G. Segregation analysis confirmed that the variants were in *trans*. For the branchpoint variant, SpliceAI predicted an acceptor gain with a high score (0.47) at position c.6480-47. A midigene splice assay in HEK293T cells revealed the inclusion of the last 47 nucleotides of intron 47 creating a premature stop codon and allowed to categorize the variant as moderately severe. Subsequent analysis revealed the presence of this variant as a second allele besides c.1958G>A p.(Arg653His) in an additional Spanish proband in a large cohort of IRD cases.

**Conclusion:** A splice-altering effect of the branchpoint variant, confirmed by the midigene splice assay, along with the identification of this variant in a second unrelated individual affected with STGD, provides sufficient evidence to classify the variant as likely pathogenic. In addition, this research highlights the importance of studying non-coding regions and performing functional assays to provide a conclusive molecular diagnosis.

## 1 Introduction

Inherited retinal dystrophies (IRDs) are a clinically complex and heterogenous group of visual impairment disorders that can result in progressive vision loss and eventual blindness. Today, there are more than 280 genes that have been associated with IRDs (https://web.sph.uth.edu/RetNet/home.htm).

*ABCA4* is among the most commonly mutated genes associated with IRDs (Quazi and Molday, 2014; Perea-Romero et al., 2021). The gene encodes the ATP-binding cassette transporter type A4 (ABCA4), a retina-specific protein that is expressed in the outer segments of photoreceptors and functions to process the metabolites of vitamin A in the visual cycle (Sun et al., 1999; Tsybovsky et al., 2010; Molday, 2015). Dysfunction of ABCA4 leads to the accumulation of cytotoxic products (lipofuscin) in the photoreceptors and retinal pigment epithelium and can manifest in different phenotypes such as Stargardt disease type 1 (STGD1), fundus flavimaculatus, cone–rod dystrophy (CRD), and pan-retinal CRD (Allikmets et al., 1997; Cremers et al., 1998; Rozet et al., 1999; Maugeri et al., 2000; Cremers et al., 2020).

A genotype–phenotype correlation model was proposed to explain the wide range of phenotypes associated with biallelic pathogenic variants in *ABCA4*. This genotype–phenotype correlation model links the residual activity of the ABCA4 protein to the severity of retinal dystrophy (van Driel et al., 1998; Maugeri et al., 1999). The model categorizes variants as deleterious (no activity; null allele), severe, moderately severe, or mild (also mentioned as hypomorphic). Some mild variants, such as c.5603A>T (p.(Asn1868Ile)), when in *trans* with a severe variant, show incomplete penetrance (Runhart et al., 2018). Patients with two severe variants or null alleles present with pan-retinal CRD, while a severe variant combined with a moderately severe variant results in CRD. On the other hand, a combination of a severe and mild variant or two moderately severe variants gives rise to classic STGD1 (Cremers et al., 2020). A combination of a severe variant with a mild-incomplete penetrant variant most often results in late-onset STGD1.

More than 2,300 unique variants have been reported for *ABCA4* (http://www.lovd.nl/ABCA4) since being first reported in 1997 (Allikmets et al., 1997; Cornelis et al., 2017; Cornelis et al., 2022; Cornelis et al., 2023). A wide variety of causative genetic defects have been reported that include missense and nonsense variants, indels, canonical, non-canonical splice site defects, and deep-intronic variants. Approximately 25% of these variants affect RNA splicing by altering one or more of the key splicing elements (Khan et al., 2020; Corradi et al., 2022).

The splicing process is a complex phenomenon that involves a large number of proteins with various interactions between the *cis* and *trans* elements. The *cis* elements are the DNA sequences that define exons, introns, and other regulatory sequences necessary for proper splicing. The branchpoint sequence (BPS) is one of the key cis-acting elements, together with the canonical 5ʹ splice donor site (SDS) and the canonical 3ʹ splice acceptor site (SAS). The BPS is a short degenerate motif typically located upstream from the SAS and followed by a cytosine- and thymidine-rich sequence called the polypyrimidine tract. The BPS is recognized by proteins involved in the formation of the spliceosome complex and is thought to play a key role in positioning the spliceosome at the correct location for efficient splicing. Additionally, there are exonic and intronic regulators that act as enhancers or silencers (Anna and Monika, 2018; Tang et al., 2020).

In recent years, causative variants in *cis* elements, which include the BPS, have been described to disrupt pre-mRNA splicing, leading to dysfunctional proteins and retinal disease (Leman et al., 2020; Corradi et al., 2022; Fadaie et al., 2022; Reurink et al., 2023). *In silico* prediction tools can identify potential splicing variants and their putative effect, but lack accuracy for novel intronic variants outside of the splice site consensus sequence (Ohno et al., 2018; Rowlands et al., 2021). However, the introduction of SpliceAI provides an accurate prediction for deep-intronic variants (Riepe et al., 2021). According to the ACMG guidelines, however, these tools only serve as indicators of splicing aberrations and are not standalone evidence for determining pathogenicity (Richards et al., 2015). Experimental studies, such as the minigene splice assays, are crucial for determining the functional impact of variants that affect RNA splicing and increasing our knowledge of these variants. These studies enable accurate classification of the severity of variants and, together with the genotype–phenotype correlation model, provide conclusive clinical diagnoses, appropriate genetic counseling, and information about disease progression.

In this study, we describe the pathogenicity of a near-exon aberrant RNA (NEAR) splice variant, c.6480-35A>G in *ABCA4*, which alters the BPS upstream of exon 48 at its most critical position. We explore the effect of c.6480-35A>G using a midigene splice assay and show the relevance of assessing branchpoint motif regions in IRDs.

## 2 Materials and methods

### 2.1 Clinical evaluation

The participants were clinically examined by an experienced ophthalmologist. The clinical diagnoses were based on ophthalmological examinations, which included assessment of visual acuity, detailed fundoscopic examination, fundus photography, fundus autofluorescence (FAF), and optical coherence tomography (OCT), and electrophysiological evaluations, which included full-field flash electroretinography (ERG) and multifocal ERG, following the International Society for Clinical Electrophysiology of Vision standards (McCulloch et al., 2015).

All procedures performed in this study involving human participants received approval from the ethical standards of the Ethics Committee for Drug Research in the Basque Country, Spain (CEIm-E), and the Ethics Committee of Fundación Jiménez Díaz University Hospital (CEIm-FJD) and were performed in accordance with the 2013 Declaration of Helsinki or comparable ethical standards. Prior to this study, informed consent was obtained from all participants or their legal representatives.

### 2.2 Whole genome sequencing

A proband was diagnosed with STGD1 at the Donostia University Hospital and without a previous genetic diagnosis. To identify the genetic defect for this individual, whole genome sequencing (WGS) was performed. DNA was provided by the Basque Biobank (www.biobancovasco.org) and was processed following standard operation procedures. WGS was performed by BGI on a BGISeq-500 using a 2 × 150 base pairs (bp) paired-end module, with a minimal median coverage per genome of 30-fold. The Burrows–Wheeler Aligner V.0.7814 (Li and Durbin, 2009) was used to map the WGS data to the human genome build GRCh38/hg38.

Single-nucleotide variants (SNVs) and small indels (<50 bp) were called using the Genome Analysis Toolkit HaplotypeCaller (McKenna et al., 2010). The SNVs and indels were annotated using an in-house developed pipeline based on Variant Effect Predictor (VEP V.104) and GENCODE V.34 basic gene annotations. Annotations included chromosomal location and position, reads, percent of variation, variant type (deletion, insertion, and substitution), gene component (e.g., intron, exon, splice site, 5′-UTR, 3′-UTR, and intragenic), protein effect (e.g., missense, synonymous, frameshift, and in-frame), various *in silico* prediction scores (e.g., CADD_PHRED, REVEL, and SpliceAI), Gene Ontology description, gene and disease OMIM description, gene regulation, expression data, and population frequency databases (gnomAD and in-house variant frequency whole exome sequencing/WGS data), among others.

Structural variants (SVs) were called using the Manta structural variant caller (Chen et al., 2016), based on read-pair signals (split reads and discordant read pairs) and read-depth signals (copy number changes), and the default parameters were used. The copy number variants (CNVs) were called using the Canvas Copy Number Variant Caller (Roller et al., 2016), based on read-depth evidence, and the default parameters were used. SVs and CNVs were annotated using an in-house developed pipeline based on ANNOVAR and GENCODE V.34 basic gene annotations. Annotations included chromosomal location and position, zygosity, type (e.g., deletion, duplication), gene overlap and component (e.g., intronic, exonic, intragenic), gene and disease OMIM description, gene boundary start and end, percentage overlap, and frequency of population frequency databases (1000 Genomes and in-house variant frequency SV data), among others.

### 2.3 Variant prioritization and selection

The WGS data were filtered and prioritized in two steps. First, an automatized in-house pipeline in RStudio V.4.1.3 (RStudio Team, 2020) was used, followed by a manual prioritization of the remaining variants. The SNVs and indels, from coding and non-coding regions, were filtered on the basis of a minor allele frequency of <1% in the gnomAD database v.2.1 (Karczewski et al., 2020) and an in-house variant frequency in the whole exome sequencing/WGS database from Radboudumc, which included 708 control samples. Nonsense, stop- or start-codon altering, frameshift, in-frame, missense, and (canonical) splice site variants were selected for detailed interrogation. Missense variants were prioritized based on score thresholds of different *in silico* prediction tools: CADD_PHRED (range: 1–99; predicted pathogenic ≥15) (Rentzsch et al., 2019) and REVEL (range: 0–1; predicted pathogenic ≥0.3) (Ioannidis et al., 2016). All coding and non-coding variants, were filtered on the splice predicting tool SpliceAI delta score (range: 0–1; predicted pathogenic ≥0.2) (Jaganathan et al., 2019) for gain or loss of a SDS or SAS. The Alamut™ Visual Plus 1.4 software was used as a visual aid to identify the position in which SpliceAI delta scores were predicted and characterize the genomic context of the variant, such as BPSs. Coding SVs and CNVs were filtered based on a minor allele frequency of <1% in the 1000 Genomes database (Zheng-Bradley et al., 2017). Inversion and duplication events were only considered when disrupting an IRD-associated gene (https://web.sph.uth.edu/RetNet/home.htm, accessed 1/11/2022), i.e., when at least one breakpoint was located within the respective gene. Compound heterozygous or homozygous candidate variants (recessive) or heterozygous candidate variants (dominant) that overlapped with IRD-associated genes were selected for validation and segregation analysis.

### 2.4 Midigene splice assay

The interrogation of a putative causal splice site variant, c.6480-35A>G in *ABCA4* (GenBank: NM_000350.2), was carried out using a midigene splice assay. A previously created wild-type midigene construct (BA29) was used that contained *ABCA4* exons 46–48 in the pDONR201 vector (Invitrogen) using Gateway cloning (Sangermano et al., 2018). The splice assay was performed in accordance with the previously described protocol (Sangermano et al., 2018; Corradi et al., 2022). In short, a construct harboring the c.6480-35A>G variant was generated through site-directed mutagenesis from the wild-type midigene construct followed by Gateway cloning. Subsequently, the wild-type and mutant constructs were transfected separately in HEK293T (Human Embryonic Kidney, ATCC# CRL-3216) cells. Transfection of the mutant construct was performed in duplicate using polyethylenimine (PEI) as a transfection reagent. After 48 h of incubation, RNA was collected using the NucleoSpin RNA kit (MACHEREY-NAGEL, Düren, Germany), and the transcripts were analyzed by reverse transcription polymerase chain reaction (RT-PCR) with primers located in exons 46 and 48, using the iScript cDNA Synthesis Kit (Bio-Rad, Hercules, CA, United States). RT-PCR was performed as follows: 2 min at 94°C, followed by 35 cycles of 30 s at 94°C, 30 s at 58°C, and 5 min at 72°C, with a final extension step of 2 min at 72°C. The RT-PCR product mixture was separated on a 2% agarose gel, and the product was verified by Sanger sequencing. Details on the primers used for mutagenesis, RT-PCR, and Sanger sequencing are presented in Supplementary Table S1. After agarose gel electrophoresis, a semi-quantification analysis of the ratios between different RNA products was carried out using the Fiji software (Schindelin et al., 2012) as previously described (Corradi et al., 2022).

## 3 Results

### 3.1 Clinical findings

**FIGURE 1:**
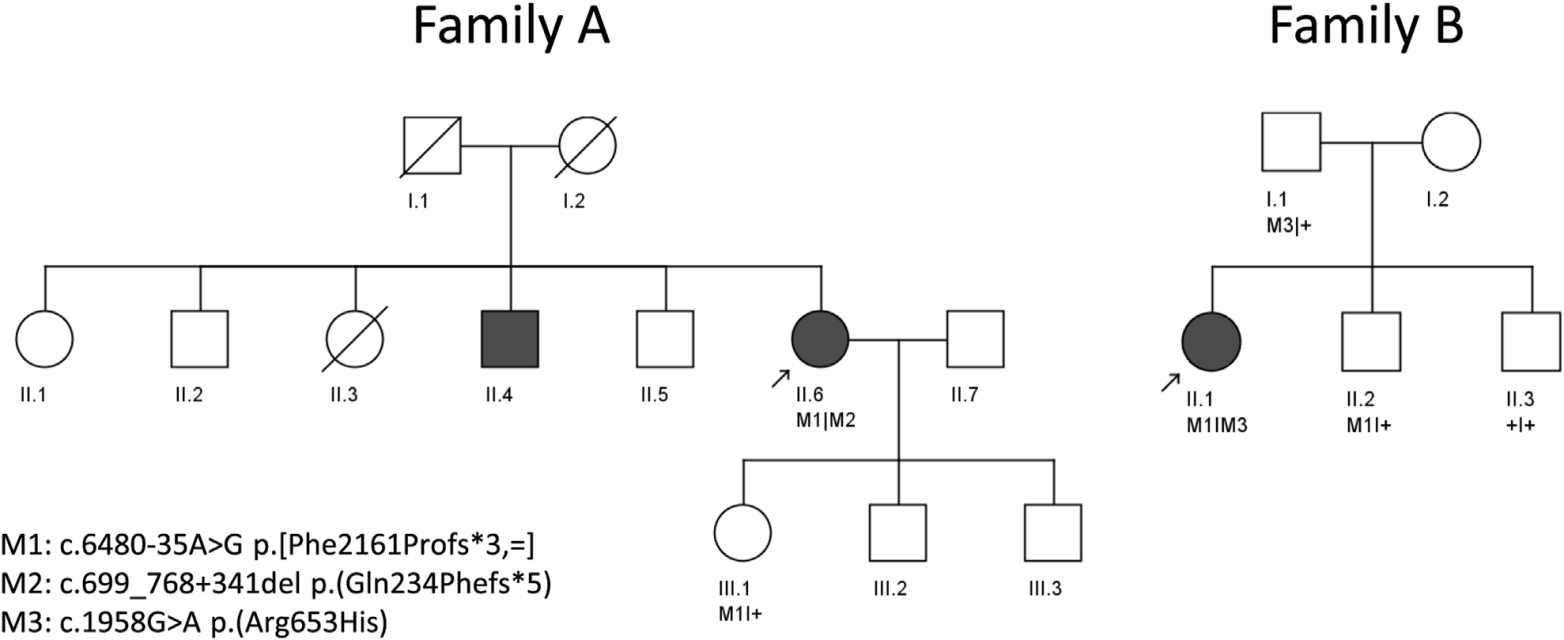
Pedigrees of two unrelated individuals analyzed in this study. Arrows indicate the proband in each family.

**FIGURE 2:**
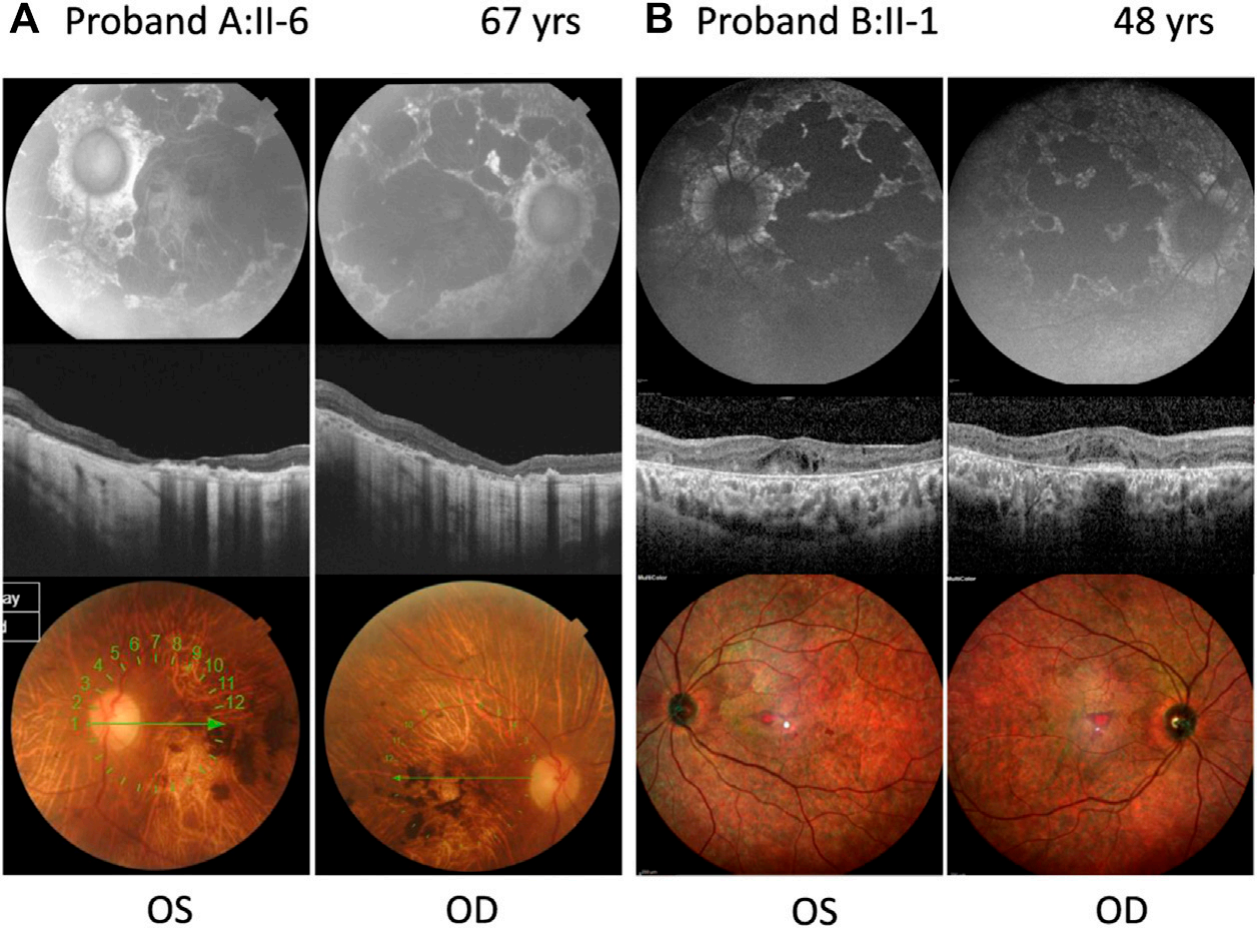
Ophthalmic features of compound heterozygous retinopathy cases carrying c.6480-35A>G. Fundus autofluorescence (upper panel), OCT (middle panel), and color fundus (lower panel) for left (OS) and right (OD) eyes. **(A)** Proband A:II-6 carrying c.699_768+341del p.(Gln234Phefs*5) as the second allele. **(B)** Proband B:II-1 carrying c.1958G>A p.(Arg653His) as the second allele. Years, yrs.

Pedigrees of the two studied families of Spanish origin with candidate variants in *ABCA4* are shown in Figure 1. Both probands presented with advanced STGD1 (Family A and B). An overview of the clinical characteristics is provided in Figure 2 and Table 1.

**TABLE 1:** Clinical characteristics of *ABCA4* retinopathy probands with c.6480-35A>G.

Patient ID (institution ID)	Sex	Age (yrs)	Initial symptom, age (yrs)	Initial clinical diagnosis	Age at last examination (yrs)	Visual acuity (logMAR)		Foveal photoreceptors	Fundus autofluorescence abnormalities	Electroretinogram
						OS	OD			
**A:II-6** (RP145)	F	67	21	Stargardt disease	67	1.3	1.3	Loss of photoreceptors in foveal region	Patchy areas of hypo-autofluorescence in the posterior pole. Preservation of peripapillary regions	Severe cone and rod responses (57 years)
**B:II-1** (MD-1378)	F	51	41	Stargardt disease	48	0	0	Loss of photoreceptors in perifoveal region	Area of RPE atrophy and hypo-autofluorescence	Severe alterations of rod responses and moderate–severe alterations in cones

F, female; OD, right eye; OS, left eye; RPE, retinal pigment epithelium; yrs, years.

Proband A:II-6 (Figure 2A) had an onset of visual complaints at the age of 21 years and was diagnosed with STGD1, with a mean visual acuity of 1.8 logMAR at the age of 67 years. The FAF images showed patchy areas of hypo-autofluorescence in the posterior pole with peripapillary sparing. OCT revealed atrophy of the outer retina layers and a loss of photoreceptors in the foveal region. The fundus images showed macular, posterior pole, and mid-peripheral chorioretinal atrophy, without flecks and with bone spicules in the mid-periphery. ERG of the proband at the age of 53 years showed severely altered cone and rod responses (extinguished in the right eye). The abnormal cone and rod responses progression to a CRD diagnosis. Unfortunately, we had no access to any ophthalmologic data nor was the DNA of the affected brother available to confirm segregation.

Proband B:II-1 (Figure 2B) was diagnosed with STGD1 at the age of 41 years, through FAF, OCT, and fundus examination (Supplementary Figure S1). An ophthalmic examination at the age of 48 years revealed a normal visual acuity (0 logMAR), with severe constriction of the visual field, suggesting foveal sparing. Multicolor and FAF images showed diffuse retinal atrophy and hypo-autofluorescence involving the whole retinal posterior pole and mid-periphery. The OCT OD image showed the foveolar area with identifiable external retinal layers in less than the central 100 microns, and the OCT OS image showed severe disturbances in the foveal photoreceptors line and the presence of cystic spaces. ERG revealed moderate–severe alterations in cones and severe alterations of rod responses, suggesting advanced STGD1.

### 3.2 Identification of c.6480-35A>G in *ABCA4* by whole genome sequencing

To study potential candidate variants in IRD-associated genes, we performed WGS in proband A:II-6. This case remained genetically unexplained after previous genetic testing through a gene panel containing 316 IRD-associated genes (Ezquerra-Inchausti et al., 2018) and CGH arrays. In total, 5,055,143 SNVs/indels were detected through WGS. From 151,997 variants with a gnomAD AF ≤1% in the general population, 559 variants were considered potentially pathogenic, as they met our inclusion criteria as a nonsense, stop- or start-codon altering, frameshift variant, in-frame insertion or deletion, potentially pathogenic missense, and (canonical) splice site variants. From 559 variants, no homozygous variants were identified, and 16 heterozygous SNVs/indels were located in IRD-associated genes. Next, 10,536 SVs and 1,307 CNVs were called by Manta and Canvas, respectively, of which 689 SVs and 255 CNVs overlapped a coding region and had an AF ≤1% in the 1000 Genomes database. From 93 SV/CNVs spanning an IRD-associated gene, only one SV had at least one breakpoint within an IRD-associated gene. Collectively for the SNV and CNV/SV data, this yielded one single compound heterozygous situation in the *ABCA4* gene.

**FIGURE 3:**
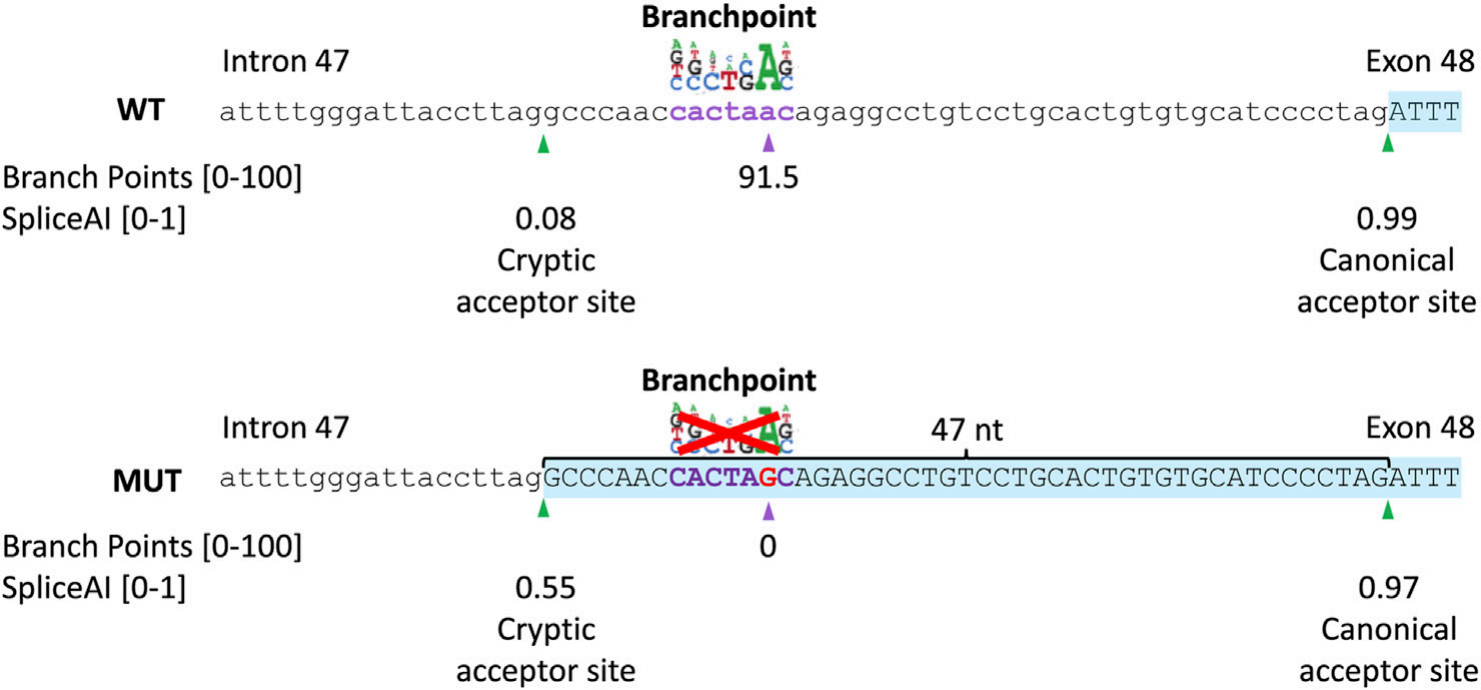
*In silico* prediction scores of the c.6480-35A>G variant. Schematic representation of the intron 47–exon 48 boundary sequence of *ABCA4*, branchpoint, and splice prediction in the wild-type (WT; upper panel) and c.6480-35A>G (MUT; lower panel) situation. The branchpoint algorithm predicts the abolishment of the branchpoint. SpliceAI predicts a 0.47 increase in the probability of activation of a cryptic acceptor site in intron 47 (47 nt upstream of the canonical acceptor site).

A novel intronic variant c.6480-35A>G (chr1(GRCh38):g.93998145T>C) was observed, which was absent from the control populations in gnomAD and located outside the SAS consensus sequence, which implies a potential impact on additional splicing elements like the BPS. Moreover, c.6480-35A>G alters the recognition score for the branchpoint algorithm, embedded in Alamut Visual Plus (range: 0–100) from 91.48 in the wild-type to zero in the mutant as a result of the removal of the branchpoint “A” nucleotide from the motif (Figure 3 upper panel). The c.6480-35A>G variant has little to no effect on splicing of the canonical SAS, as shown by the Splice score prediction algorithms. For the cryptic SAS at position c.6480-47, NNSPLICE predicts a 4.9% higher score than the wild-type situation, GeneSplicer predicts a 12.1% lower score, and no changes in the score values of MaxEntScan and SpliceSiteFinder-like. Nevertheless, the SpliceAI algorithm predicts a significant strengthening of the cryptic SAS in intron 47 (47 nt upstream of the canonical SAS) with delta scores of 0.47 and a loss of the canonical SAS of exon 48, with a delta score of 0.02 (Figure 3 lowel panel). The SV consisted of a deletion of 411 bp in *ABCA4* spanning 70 bp of exon 6 and 341 bp of intron 6, c.699_768+341del; p.(Gln234Phefs*5), as was previously identified in the Spanish population (Del Pozo-Valero et al., 2020).

The clinical/whole exome sequencing data from a Spanish cohort (the Fundación Jiménez Díaz cohort) of 52 probands with Stargardt disease and 26 probands with CRD, and one likely pathogenic or pathogenic variant in *ABCA4* were investigated for the presence of c.6480-35A>G. This analysis revealed a second case, proband B:II-1. This individual carried the c.1958G>A; p.(Arg653His) variant, a known likely pathogenic variant. Segregation analysis confirmed that in both families, the variants were compound heterozygous as available unaffected relatives carried one of these two variants in a heterozygous state. Additional analysis of the whole exome sequencing data of 1,935 genetically unexplained cases did not reveal probands carrying the variant of interest.

### 3.3 Midigene splice assay results

**FIGURE 4:**
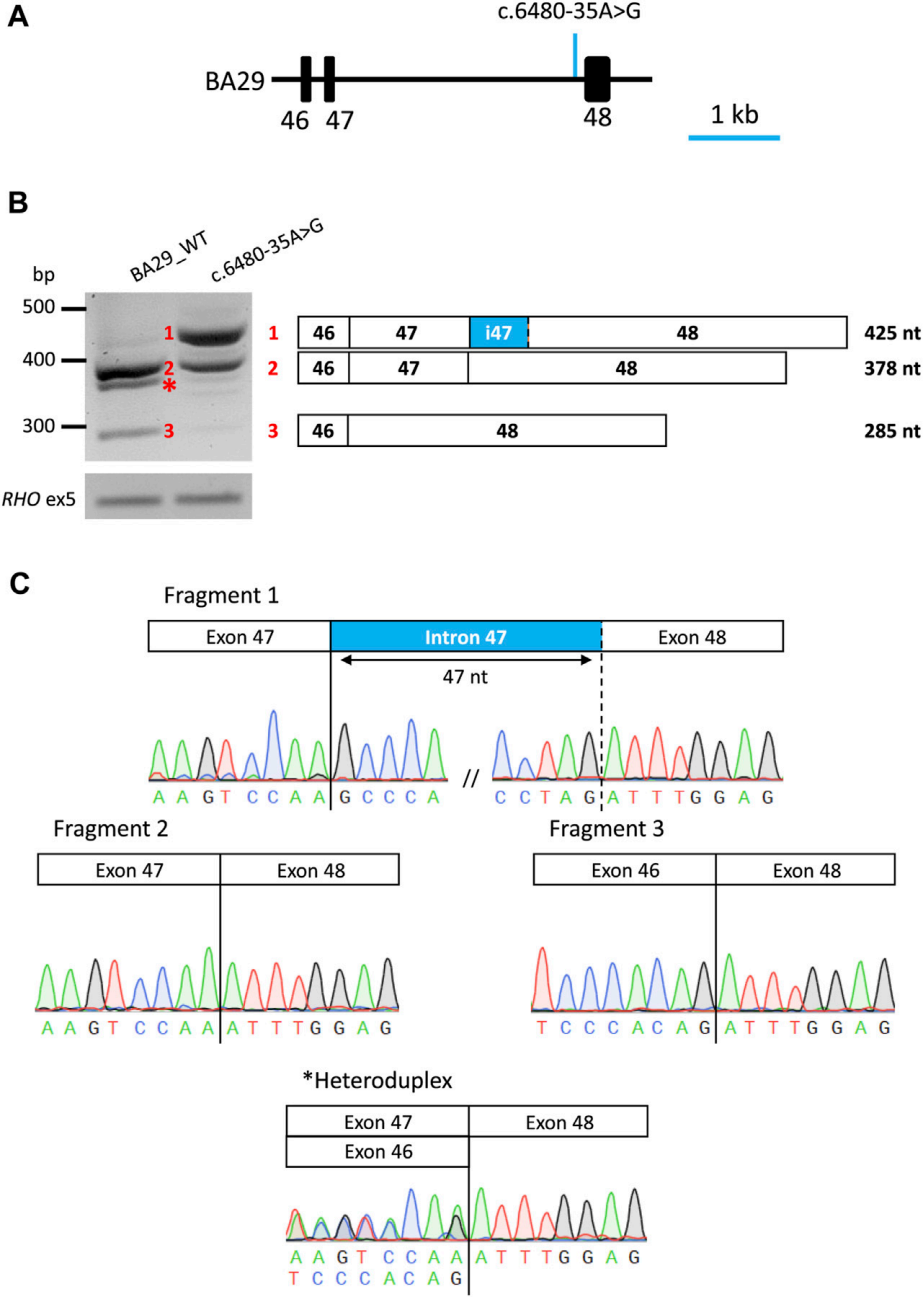
Overview of midigene assay results of variant c.6480-35A>G in HEK293T cells. **(A)** Schematic representation of wt midigene (BA29_WT) where the position of the variant is indicated by an arrow. **(B)** Gel image of RT-PCR products of wild-type and mutant constructs. The rhodopsin exon 5 (*RHO* ex5) RT-PCR was used as a control for transfection efficiency. Schematic representation of the three RT-PCR products identified in the gel. Wt midigene reveals the expected 378 nt wt fragment (Fragment 2) and the exon 47 skipping fragment (Fragment 3). Mutant midigene reveals a partial intron 47 inclusion of 45 nt 5ʹ (Fragment 1) and 30.4% of the remaining wt fragment (Fragment 2). Fiji software was used for a semi-quantification of the fragments in the mutant construct. **(C)** Sanger sequence analysis of the RT-PCR fragments. The chromatograms show the breakpoints in all fragments. * Heteroduplex fragment.

To assess pathogenicity of c.6480-35A>G, a midigene splice assay was performed (Figure 4). HEK293T cells were transfected either with a wild-type midigene construct spanning *ABCA4* exon 46–48 or a mutant construct carrying c.6480-35A>G within the same region.

After RNA isolation and RT-PCR analysis of the individual midigenes, a predicted 378 nt fragment was detected corresponding to the *ABCA4* wild-type mRNA, for the wild-type. In addition, a 285 nt fragment showing exon 47 skipping of *ABCA4* mRNA was observed, resulting in an in-frame deletion of 31 amino acid residues (p.(Ser2129_Lys2160delinsArg)). In the mutant midigenes, a 425 nt fragment (∼67% of the PCR product) was observed in addition to the wild-type fragment (∼30% of the PCR product) and a minimal contribution of the exon 47 skipping event. Sanger sequencing verified that the 425 nt fragment corresponded to the inclusion of the last 47 nt of intron 47 at the 5′ start of exon 48, likely due to the activation of a cryptic SAS at position c.6480-47, as predicted by SpliceAI. This inclusion results in a frameshift that includes a premature stop codon along with conventionally spliced mRNA (p.[Phe2161Profs*3,=]).

## 4 Discussion

In this study, we identified a novel pathogenic branchpoint variant, c.6480-35A>G, in *ABCA4* using WGS and a subsequent dedicated midigene splice assay. The variant abolishes the putative branchpoint of intron 47, leading to a 47 nt retention of intron 47 due to the activation of a cryptic SAS. The severity of a variant can be determined by the percentage of correct RNA remaining in the midigene splice assay in HEK293T cells (Sangermano et al., 2018; Cremers et al., 2020). The midigene splice assay revealed a moderately severe (range: 20%–40% normal RNA, FPMC, unpublished data) effect for c.6480-35A>G as 30.4% of the wild-type fragment remained alongside the mutant fragments after semi-quantification analysis. This knowledge is important for consideration of disease presentation and prognosis as the residual activity of the ABCA4 protein correlates with the severity of *ABCA4*-associated retinopathy.

The genotype–phenotype correlations in our study cohort also suggest the effect of variant c.6480-35A>G as moderately severe (likely pathogenic based on the ACMG classification). Different phenotypes were observed in the two probands carrying the variant c.6480-35A>G. In particular, A:II-6, who carries the variant in *trans* with c.699_768+341del; p.(Gln234Phefs^∗^5), showed a more severe phenotype associated with STGD1 and additional degeneration of the cone and/or rod photoreceptor cells over time, leading to a phenotype that more closely resembles CRD. The variant c.699_768+341del is a null variant, is classified as severe and was previously associated with both STGD1 and CRD (Del Pozo-Valero et al., 2020). According to our data, individuals with STGD1 who have one severe variant in combination with one moderately severe variant may progress to CRD. Therefore, additional ophthalmologic assessments, which include ERG, should be taken into account. B:II-1, who carries c.6480-35A>G in *trans* with c.1958G>A; p.(Arg653His), showed a milder phenotype associated to late-onset STGD1. The variant c.1958G>A has been previously associated with STGD1 (Jiang et al., 2016; Sung et al., 2019; Garces et al., 2020; Ma et al., 2021). Moreover, it has been previously classified to have a mild/moderate effect by Garces et al. (2020) and a severe effect by Cornelis et al. (2022). The phenotypic assessment of the proband B:II-1 suggests a moderately severe effect of c.1958G>A.

Only recently, the first BPS variants associated with IRDs have been identified in *BBS1* (Fadaie et al., 2022) and *ABCA4* (Corradi et al., 2022), while the recognition of the BPS is crucial for the formation of the lariat structure prior to intron excision from pre-mRNA. Identification of pathogenic BPS variants may be hampered by the challenges of recognition of the BPS sites due to its localization and the conserved motif of BPSs. While the majority of BPSs have been identified in a window of 18–44 nt upstream of the SAS, BPSs located up to 400 nt away from the SAS have also been found (Gooding et al., 2006). The limited number of experimentally validated wild-type and mutated BPSs has posed challenges in developing effective tools to predict the impact of variants upstream of SASs. Alamut Visual Plus prediction tools such as NNSPLICE indicated an increase of 4.9% for the cryptic SAS at position c.6480-47, while GeneSplicer indicated a reduction of 12.1% at position c.6480-47. However, the branchpoint prediction incorporated in Alamut Visual Plus showed a predictive score for the wild-type (91.5), which is completely abolished in the mutant. Moreover, SpliceAI accurately predicted partial intron retention as confirmed by our *in vitro* splice assay, which highlights that SpliceAI proves to be effective in predicting the impact of BPS variants on splicing.

To assess the effect of the variant, *in vitro* splice assays using HEK293T cells have been previously shown to accurately recapitulate splice defects affecting consensus splice site sequences at the exon–intron junctions, as well as most variants that generate new splice sites or enhance cryptic splice sites in introns, leading to pseudo-exon inclusion, exon elongation, or intron retention (Sangermano et al., 2018; Bauwens et al., 2019; Valero et al., 2019; Westin et al., 2021; Viering et al., 2022). The midigene assay in this study effectively demonstrated that the c.6480-35A>G variant resulted in an altered splicing pattern. However, we also observed exon 47 skipping in wild-type mRNA. It remains to be determined whether this is a natural exon skipping event or an artifact due to the lack of retina-specific factors in HEK293T cells and the artificial nature of the midigene system. Therefore, the analysis of retina mRNA, photoreceptor precursor cells, or retinal organoids generated from induced pluripotent stem cells derived from patient offer a more relevant context for observing the variant’s effects (Vig et al., 2020; Mullin et al., 2021).

To date, there are no FDA-approved therapies for *ABCA4*-associated retinopathy, but several experimental treatments are being studied. Antisense oligonucleotide (AON)–based therapeutic strategies have shown effectiveness in modulating splicing and obtaining correct transcripts in *ABCA4* in several studies (Albert et al., 2018; Garanto et al., 2019; Sangermano et al., 2019; Tomkiewicz et al., 2021; Kaltak et al., 2023). Nevertheless, the use of AONs to treat the effects of c.6480-35A>G could potentially result in its binding to the region upstream of the canonical SAS that may disrupt regulatory motifs and the binding of auxiliary splice proteins. Additionally, recent studies have shown the efficiency of the CRISPR/Cas9 system in correcting variants in the *ABCA4* gene without off-target genomic alterations (De Angeli et al., 2022; Siles et al., 2023). These are promising areas of research that could potentially lead to effective treatments for *ABCA4*-associated retinopathy, but more research is required to determine their safety and effectiveness in clinical trials.

In conclusion, we have identified a novel variant in *ABCA4*, c.6480-35A>G, which disrupts a predicted branchpoint, leading to inclusion of 47 nt in the mRNA resulting in protein truncation. This variant was observed in two unrelated individuals of Spanish descent. We determined that c.6480-35A>G can be classified as moderately severe. In combination with a deletion with a severe effect, it underlies STGD1 progressing to CRD in proband A:II-6. In proband B:II-1, this variant, in *trans* with a moderately severe missense variant, led to late-onset STGD1. Furthermore, this study emphasizes the significance of investigating non-coding regions and conducting functional assays to establish a better molecular diagnosis.

## Data Availability

The variant data presented in this study have been submitted to the “Global Variome shared LOVD” and it can be accessed using the url: https://databases.lovd.nl/shared/references/DOI:10.3389/fgene.2023.1234032.

## Supplementary material

SUPPLEMENTARY FIGURE S1 Ophthalmic features of proband B:II-1 at two ages. Fundus autofluorescence (upper panel), OCT (middle panel), and color fundus (lower panel) for left (OS) and right (OD) eyes. **(A)** Proband B:II-1 at 41 years. **(B)** Proband B:II-1 at 48 years.

SUPPLEMENTARY TABLE S1 Oligonucleotides employed in this study.
