# Supplementary material for: ABCA4 c.6480-35A>G, a novel branchpoint variant associated with Stargardt disease
Source: Front Genet. 2023 Sep 7;14:1234032. doi: 10.3389/fgene.2023.1234032 (PMC10539688; doi:10.3389/fgene.2023.1234032)
Supplement: Supplementary file 1 [file Table1.pdf]

SUPPLEMENTARY TABLE S1

Oligonucleotides employed in this study.

Name	Sequence (5' to 3')	Genomic position (hg38)	Product size (bp)
Mut_c.6480-35A>G_Fwd	CAGGCCTCTGCTAGTGGTTGGGCCTAAGG	-	6,457
Mut_c.6480-35A>G_Rev	CCTTAGGCCCAACCACTAGCAGAGGCCTG	-	
BA29_Fwd	ATCAGAGAAGGGAGGGCTGT	Chr1:94001020	425
BA29_Rev	GTCCAGTGTGGTCTGTGTGAC	Chr1:94463420	
*RHO*_exon5_Fwd	ATCTGCTGCGGCAAGAAC	Chr3:129533632	140
*RHO*_exon5_Rev	AGGTGTAGGGGATGGGAGAC	Chr3:129533752	
