# Supplementary figures and images for: ABCA4 c.6480-35A>G, a novel branchpoint variant associated with Stargardt disease
Source: Front Genet. 2023 Sep 7;14:1234032. doi: 10.3389/fgene.2023.1234032 (PMC10539688; doi:10.3389/fgene.2023.1234032)

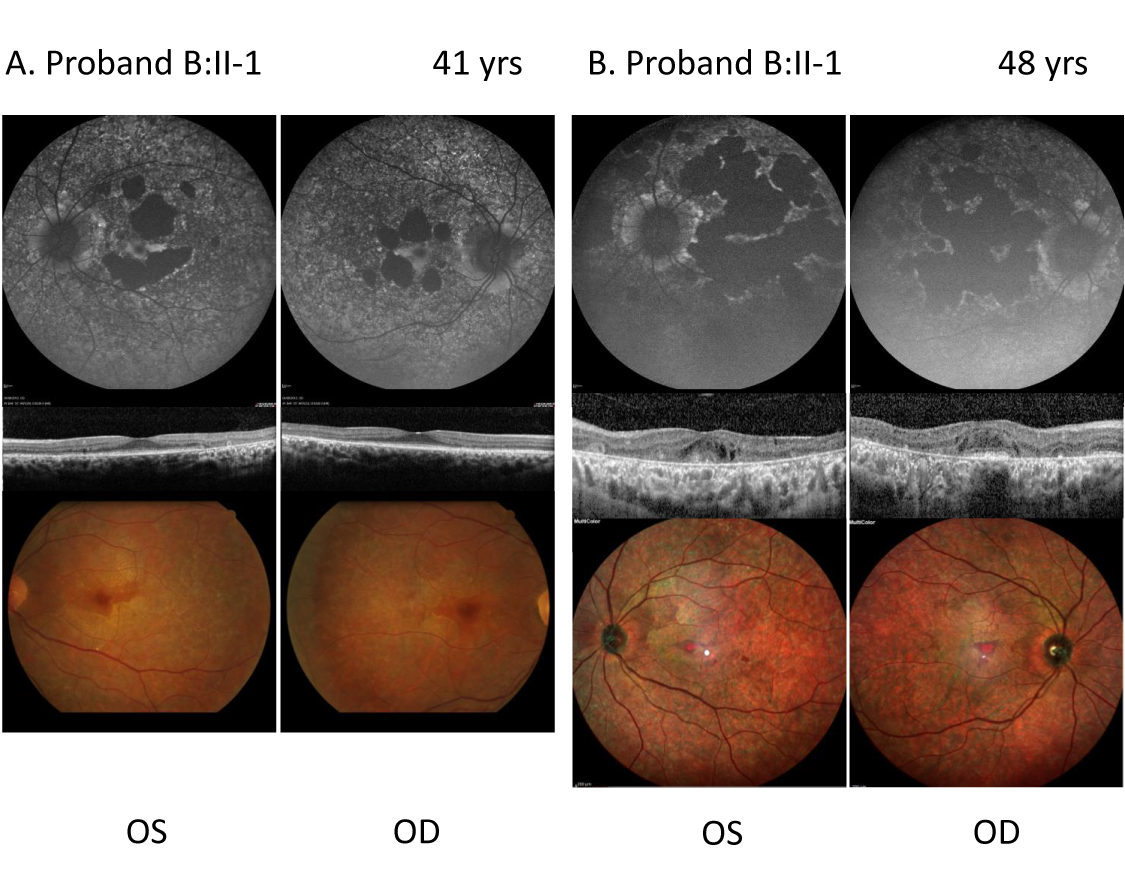

Supplement: Supplementary file 2 [file Image1.tif]
